# Sea ice phenology and primary productivity pulses shape breeding success in Arctic seabirds

**DOI:** 10.1038/s41598-017-04775-6

**Published:** 2017-07-03

**Authors:** Francisco Ramírez, Arnaud Tarroux, Johanna Hovinen, Joan Navarro, Isabel Afán, Manuela G. Forero, Sébastien Descamps

**Affiliations:** 1 Estación Biológica de Doñana (EBD-CSIC), Sevilla, Spain; 2 Norwegian Polar Institute, Fram Centre, 9296 Tromsø, Norway

## Abstract

Spring sea ice phenology regulates the timing of the two consecutive pulses of marine autotrophs that form the base of the Arctic marine food webs. This timing has been suggested to be the single most essential driver of secondary production and the efficiency with which biomass and energy are transferred to higher trophic levels. We investigated the chronological sequence of productivity pulses and its potential cascading impacts on the reproductive performance of the High Arctic seabird community from Svalbard, Norway. We provide evidence that interannual changes in the seasonal patterns of marine productivity may impact the breeding performance of little auks and Brünnich’s guillemots. These results may be of particular interest given that current global warming trends in the Barents Sea region predict one of the highest rates of sea ice loss within the circumpolar Arctic. However, local- to regional-scale heterogeneity in sea ice melting phenology may add uncertainty to predictions of climate-driven environmental impacts on seabirds. Indeed, our fine-scale analysis reveals that the inshore Brünnich’s guillemots are facing a slower advancement in the timing of ice melt compared to the offshore-foraging little auks. We provide a suitable framework for analyzing the effects of climate-driven sea ice disappearance on seabird fitness.

## Introduction

Arctic ecosystems are rapidly changing as climate warming affects these regions faster than the global average^1–3^. Among the main climate-driven environmental changes occurring in the Arctic, it has been suggested that the recent and dramatic loss of sea ice^4, 5^ is most strongly impacting the structure and functioning of polar communities^1, 6–9^. However, the actual consequences of sea ice loss on key biological processes remain largely unknown^9^. This is, in part, because polar systems, especially marine ones, are less accessible and notoriously difficult to study^7, 10^.

Since the late ‘70 s, the Arctic sea ice has decreased at a rate of 5% per decade^4, 5^. It is expected that this trend will continue well into the future, with the strongest losses predicted in the southern Arctic seas, including the Barents Sea area^7, 11–13^. These changes in the physical environment are evident in the marine food webs through phenological changes and mismatching processes^14–16^, or alterations in predator-prey dynamics with cascading impacts on their populations^6, 7, 17, 18^. However, one of the most alarming, albeit largely undocumented impact, probably concerns changes in bottom-up processes affecting the efficiency with which biomass and energy are transferred through marine food webs^8–10^.

Sea ice plays a dual role in primary production in polar seas by driving the timing of the two consecutive pulses of marine autotrophs that form the base of the Arctic food webs: sea-ice algae and pelagic phytoplankton^9^. Sea-ice algae begin to grow within and underneath the sea ice in early spring, concurrently with increasing day length and with the start of the ice melting process^19, 20^. The pelagic algal bloom, in contrast, normally occurs in open waters several weeks after ice breakup, leading to a discontinuity between sea-ice and open-water primary production^8–10, 20^. As the seasonal growth period becomes narrower at higher latitudes, it is suggested that the timing for these two blooms is probably the most essential driver of secondary production^10^. Indeed, the breeding phenology of Arctic zooplankton (mainly the copepod *Calanus glacialis*) has been shown to closely track the early sea-ice algae bloom, whereas the growth of this secondary producer’s offspring is dependent on the later bloom of pelagic phytoplankton^8, 9^. Changes in the timing of these productivity pulses may therefore result in a mismatch between zooplankton requirements and resource availability, thereby affecting zooplankton recruitment, hence the efficiency of transfer of biomass and energy to higher trophic levels^8, 9, 20^. Ultimately, this may affect some valued ecosystems services, such as fish stocks, as well as marine mammals, seabirds and other marine-dependent Arctic predators^21^. Indeed, trophic disruptions affecting marine productivity patterns^22, 23^, species distributions^7, 10^ or phenological processes^8, 9^ may lead to rapid and sensitive responses in the reproductive performance of top-predators^22^. Monitoring these reproductive parameters could therefore serve as a sensitive tool for tracking changes in the functioning of Arctic marine ecosystems on a near real-time basis, provided we are able to link the large degree of climatic variability to changes in the breeding performance of top-predators^24^.

Here, we used remotely sensed records to investigate the chronological sequence of the primary productivity pulses (i.e. sea-ice algae and pelagic phytoplankton annual blooms) affecting the transfer of biomass and energy through Arctic food webs. Satellite remote sensing has emerged as an essential tool for studying the most recent and striking trends and patterns in both physical (e.g., changes in sea ice extent phenology^6, 7^) and biological (e.g., marine productivity^22, 23^) processes in oceans worldwide. These environmental data products are now provided at a high enough spatiotemporal resolution for capturing the fine-scale spatial heterogeneity inherent to marine systems^7^, and the short-term variability typical of these seasonal environments^22, 23^. The Svalbard archipelago (Norway) provides, in turn, an ideal case study for assessing the impact of climate-driven trophic disruptions on the fitness of Arctic top-predators. This is because environmental cycles and productivity patterns in Svalbard are largely driven by sea ice phenology^8, 10^, and because this region has recently experienced one of the highest rates of sea ice loss within the circumpolar Arctic^1, 25–27^. We used remote-sensing data on sea ice extent and chlorophyll-*a* concentration (CHL-*a*) to investigate interannual changes in seasonal patterns for each of the two blooms of autotrophs forming the base of the Arctic marine food web. We then assessed whether the timing of these two pulses of primary production affects the reproductive performance of the High-Arctic seabird community from Svalbard via bottom-up processes. Finally, we framed these results within the current trends of global warming and changes in sea ice melting patterns in the Arctic^11^.

## Results

**Figure 1:**
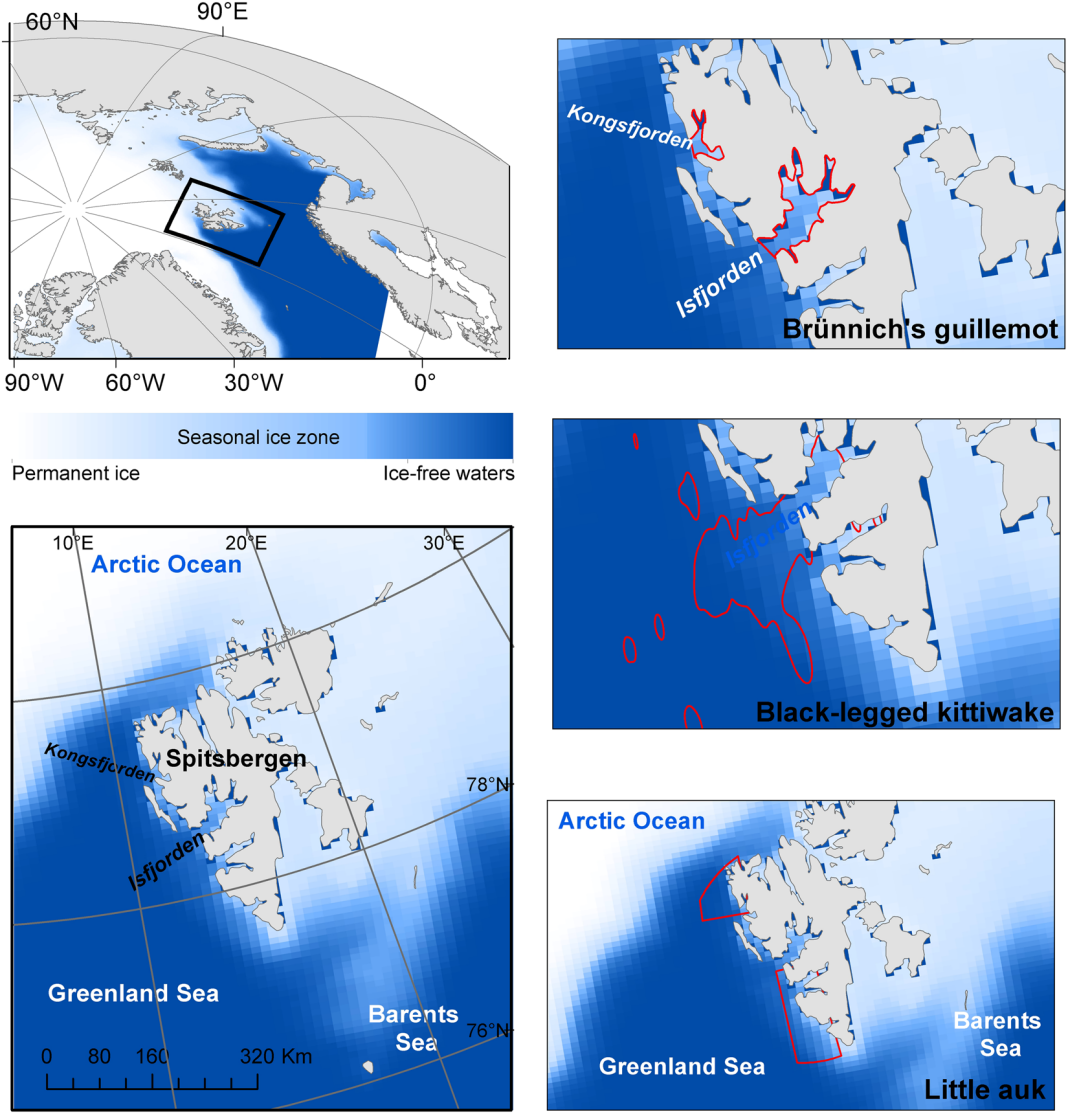
Study area and species. Based on GPS loggers and *δ* ^13^C values, we determined the most likely foraging areas for the Brunnich’s guillemots, black-legged kittiwakes and little auks breeding in the Svalbard Archipelago. These were subsequently used for investigating the sequence of environmental and biological processes driving food availability and, ultimately, seabirds’ reproductive performances. Background colors pinpoint the seasonal ice zone, based on daily images of ice extent for the 2006–2016 period (National Snow and Ice Data Center, Boulder, Colorado USA). Dark-blue in cells along the coastline indicate no data and are excluded from the analyses. Map generated with ArcGIS 10.2.1 (ESRI, Redland), www.arcgis.com. Organizational License provided by CSIC.

We focused on three of the most abundant, though ecologically diverse, seabird species breeding in the Svalbard archipelago: the Brünnich’s guillemot (*Uria lomvia*), the black-legged kittiwake (*Rissa tridactyla*) and the little auk (*Alle alle*) (see Supporting Information). The foraging areas during the summer period differed among the three seabird species (see Fig. 1 and Supporting Information). The Brünnich’s guillemot mainly foraged in coastal waters within the fjords (two main fjords, Diabasodden – Isfjorden –, and Ossian Sarsfjellet – Kongsfjorden; Supporting Information) whereas the little auks’ main foraging ground encompassed two potential offshore areas near the ice edge at the North and South of Spitsbergen (Supporting Information). All of these main foraging areas were partially covered by sea ice during the winter months, with sea ice cover >30%, a threshold previously used for delimiting the sea ice season^6, 7^. Black-legged kittiwakes, in contrast, mainly foraged in permanently ice-free waters (sea ice cover <30% throughout the annual cycle). Thus, these were likely independent from sea-ice driven productivity patterns and were excluded from the subsequent analyses.

In contrast to pelagic phytoplankton, sea ice algae concentration cannot be measured remotely^20^. However, the use of satellite-derived measurements of sea-ice cover can be used as a proxy to study large-scale patterns in sea ice algae timing. Indeed, the annual peak of sea ice cover within the seabirds’ main foraging areas was used as a surrogate for the onset of sea-ice melting and, consequently, the timing of the annual bloom of sea ice algae^10^. Seasonal patterns in CHL-*a* were used to derive open-water, pelagic algal blooms. The relative timing of, and thus the time-lag between these two pulses of primary productivity has been reported to be the essential driver of secondary production^8, 9^. This temporal lag was therefore considered as a potential driver of the efficiency with which biomass and energy are transferred from primary producers to higher trophic levels^8, 9^, ultimately affecting food abundance and top-predators’ breeding performance.

**Figure 2:**
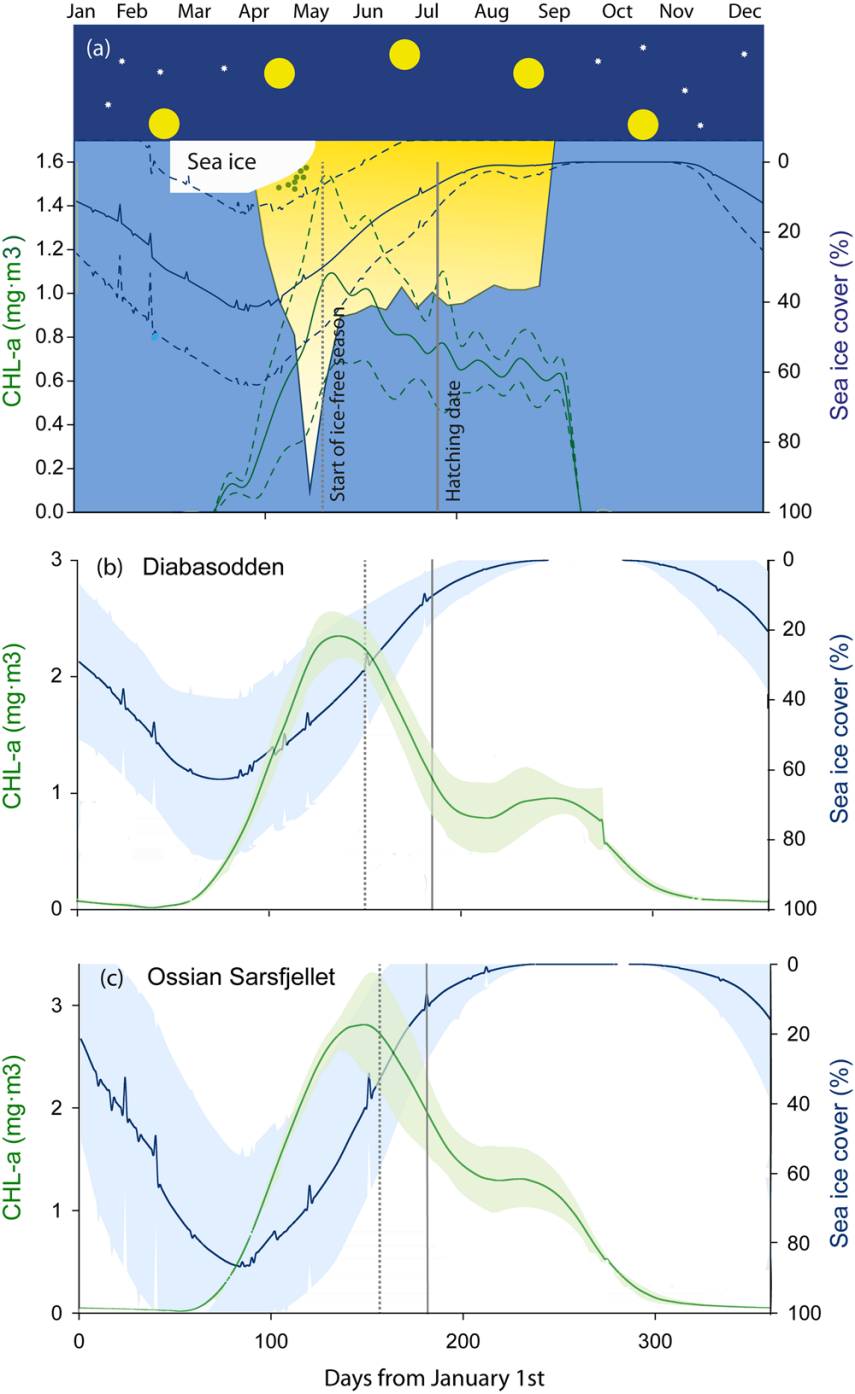
Environmental cycles in the seasonal ice zone. Principal processes regulating marine productivity within little auks (**a**) and Brunnich’s guillemots (**b**,**c**) foraging grounds (modified from Wassmann *et al*.^10^). Annually averaged (2007–2016 for little auks and 2011–2016 for Brunnich’s guillemots) seasonal trends for sea-ice extent and chlorophyll-*a* (CHL-*a*) have been used to pinpoint the annual blooms of sea ice algae and pelagic phytoplankton. KD490 (background colors in – a –) represents the capacity of light to penetrate into the water column (yellow area). 30% sea ice cover (dashed vertical line) has been used as a suitable threshold delimiting the sea ice season^6, 7^. The dark period, the height of the sun, the changing thickness of ice and the annual bloom of sea-ice algae are schematically shown in (**a**). Mean hatching dates (solid vertical lines) for each species are also represented.

On average, the annual peak of sea ice within Brünnich’s guillemot and little auk foraging grounds occurred in late March (Fig. 2). The subsequent reduction in sea ice extent continued through April, concurrently with increasing day length, and reached the 30% threshold in early and late May, respectively (Fig. 2). As the ice melting progressed and the sunlight increased, the capacity of light to penetrate into the nutrient-rich water column increased as revealed by decreasing values in KD490 (an index of light availability, see Methods) (note that KD490 data within Svalbard fjords were not available). This provided suitable conditions for phytoplankton blooming (i.e. peak in CHL-a), with peaks typically matching the start of the ice-free season (Fig. 2).

Seasonal patterns in sea ice extent and CHL-*a* varied among years within our study period. A ten to eleven week difference in the annual peak of sea ice extent was observed within little auk and Brünnich’s guillemot foraging grounds, respectively, in period 2007–2016 (little auk) and 2011–2016 (Brünnich’s guillemot). In the case of CHL-*a*, this difference accounted for 6 to 12 weeks. Accordingly, the temporal lag between the annual peaks of sea ice extent and CHL-*a* ranged from 2 to 10 weeks in the case of little auks, and from 4 to 21 weeks for Brünnich’s guillemots.

**Figure 3:**
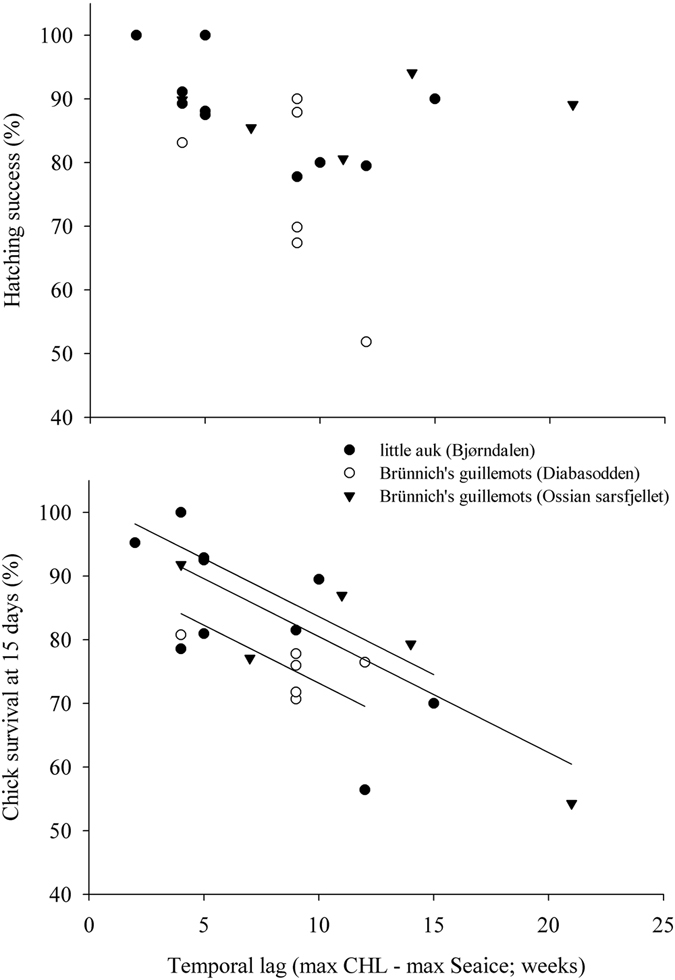
Seabirds’ breeding responses. Brünnich’s guillemot and little auk breeding responses to inter-annual changes in the temporal lag – in weeks – between the annual peaks of sea ice extent and CHL-*a*.

Seabird hatching dates were relatively constant throughout our study period (Table 1). On average, the Brünnich’s guillemot bred earlier (peak hatching in late June) than the little auk (peak hatching in early July, Table 1). In contrast, their breeding performance, in terms of hatching success and chick survival, largely varied among years (Table 1). The temporal lag between the two pulses of marine productivity was an important driver of chick survival (F_6,11_ = 4.49, p = 0.03, R^2^ = 0.69), which decreased at an overall rate of 2% per week of lag between the two pulses of marine autotrophs, regardless of the species and colony considered (see Fig. 3 and Supporting Information for model outputs). In contrast, no significant effects were observed for our estimates of hatching success (F_6,14_ = 2.4, p = 0.14, R^2^ = 0.5).

**Table 1:** Summary of main breeding parameters.

Species	Colony	Year	Hatching Date		Breeding performance (%)	
			Mean (SD)		Hatching success	Chick survival
Little auk	Bjørndalen (Isfjorden)	2007	jul-10	(4.3)	88.1	81.0
		2008	jul-09	(3.2)	89.3	78.6
		2009	jul-12	(2)	79.5	56.4
		2010	jul-08	(4.4)	100	92.5
		2011	jul-10	(5)	77.8	81.5
		2012	jul-10	(3.5)	91.1	100
		2013	jul-13	(2.7)	87.5	92.9
		2014	jul-10	(2.2)	100	95.2
		2015	jul-08	(2.7)	80	89.5
		2016	jul-06	(1.9)	90	70
Brünnich’s guillemots	Diabasodden (Isfjorden)	2011	jul-03	(3.2)	87.9	75.9
		2012	jul-07	(4.3)	67.4	70.7
		2013	jul-05	(5.5)	69.9	71.8
		2014	jul-01	(7.9)	51.9	76.5
		2015	jun-22	(10.7)	83.1	80.8
		2016	jun-26	(3.3)	90	77.8
	Ossian sarsfjellet (Kongsfjorden)	2011	jul-04	(5.3)	89.1	54.3
		2012	jul-05	(6.4)	94.1	79.3
		2013	jul-04	(6.5)	85.5	77.1
		2014	jun-29	(6.3)	80.6	87
		2015	jun-23	(9.2)	89.9	91.8
		2016	jul-02	(9.2)	89.4	73.7

Mean hatching date and standard deviation (SD), hatching success and chick survival at 15 days after hatching for the little auk and the Brünnich’s guillemot breeding in the Svalbard Archipelago.

**Figure 4:**
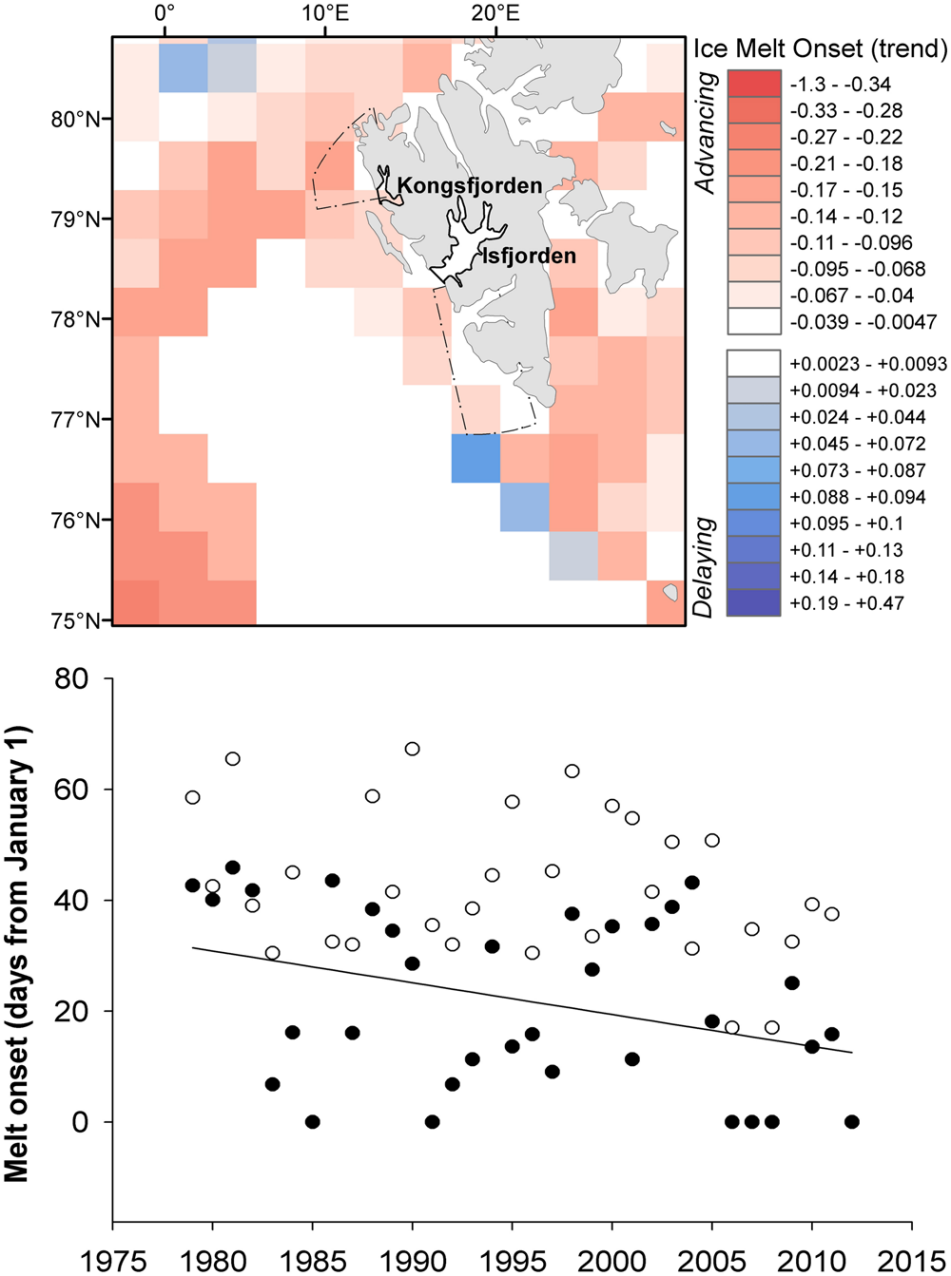
Spatially-explicit trends in the melting phenology. The map represents the slopes of the pixel-based, least squares linear regressions of snow melt onset dates for the 1979–2012 period and reveals the uneven distribution of environmental trends. The average date at which the onset of the melting process occurs annually within each seabird foraging area is also represented in a scatterplot (little auk, black dots; Brünnich’s guillemot, white dots) to show the species-specific changes in the environmental conditions that seabirds are facing. Map generated with ArcGIS 10.2.1 (ESRI, Redland), www.arcgis.com. Organizational License provided by CSIC.

Spatially-explicit information on the long-term (1979–2012) trends in the melting phenology suggested that seabirds might be facing different environmental changes according to their foraging ranges (Fig. 4). In particular, the offshore little auk feeding in the north and south of Spitsbergen have faced an advancement of 0.6 days·year^−1^ (95% CI: 0.04, 1.1) in the onset of the melting process over the last few decades (F_1,32_ = 4.8, p = 0.03, R^2^ = 0.11). A less pronounced (0.4 days·year^−1^; 95% CI: −0.9, 0.12), albeit non-significant, trend was observed for the inshore Brünnich’s guillemot (F_1,32_ = 2.5, p = 0.12).

## Discussion

Food availability during key periods such as reproduction is widely considered as one of the most important drivers of marine predator breeding performance^28^. In turn, climate driven environmental changes (e.g., increasing water temperatures) may affect marine productivity patterns, either directly through metabolic processes^29^ or indirectly through trophic disruptions caused by shifts in species distributions or abundances^10, 21, 30^ or changes in the timing of seasonal processes^9, 15, 30^. Here, we provide evidence that interannual changes in the seasonal patterns of marine productivity in the Arctic may have a cascading effect on seabirds. In particular, we showed that increasing temporal lag between sea-ice melting, i.e. the physical process driving the annual bloom of sea ice algae^10, 20^, and the bloom of pelagic phytoplankton resulted in rapidly decreasing breeding performance for little auks and Brünnich’s guillemots. The timing of these two productivity pulses is considered as an essential driver of recruitment, and hence abundance, of secondary producers^8, 9^. Therefore, climate-driven trophic disruptions seem to be an important mechanism underlying the breeding performance of Arctic seabirds and, potentially, of other marine-dependent Arctic top-predators.

Current trends towards sea ice disappearance are known to have a profound impact on the structure and functioning of sea-ice dependent Arctic ecosystems^10, 21^. Previously reported impacts on Arctic predators mostly concern changes in resource availability and interspecific relationships caused by species distribution shifts^6, 7, 17^ or changes in seasonal patterns of marine productivity^14–16^. Nevertheless, resource availability for an organism is a complex feature that encompasses food abundance and accessibility^28, 30^. In the Arctic, it has been suggested that food abundance and the efficient transfer of nutrients and energy through ice-dependent food webs largely rest on the timing of the blooms of sea ice algae and pelagic phytoplankton^10^. Previous studies on the impacts of environmentally-driven changes in food abundances have largely focused on the lower levels of Arctic food webs (primary and secondary production^8, 9^). Observed trends in the breeding performance of seabirds feeding on the seasonal ice zone (i.e., little auks and Brünnich’s guillemots) suggest, however, that these impacts may also extend through entire food webs via bottom-up processes, from primary producers up to predators. Remarkably, these trends contrasted between the two considered breeding parameters, i.e. hatching success and chick survival. This might be partially explained by the fact that maximum food requirements occur during chick rearing^22, 30, 31^. Additionally, egg synthesis can be partially sourced by resources acquired prior reproduction^32–34^, thereby mitigating food availability during the egg production period^34, 35^.

Seasonal patterns in marine productivity at high latitudes are largely constrained by sea ice cover and darkness during winter months^8, 10^. Even at the very low sunlight intensity conditions of the early spring, the onset of snow and ice melting in March (Fig. 2, see also Søreide *et al*.^9^) substantially enhances light availability within the water column and promoting ice algae growth until the sea ice substratum melts in May. Concurrently with the sea ice breakup, light availability within the water column peaked resulting in the annual bloom of pelagic phytoplankton. The timing of these physical and biological processes is largely affected by the high degree of environmental variability that characterizes Arctic ecosystems^8^. Such environmental variability is expected to become even higher within the current context of global warming^10, 21^, thus warranting more studies of the mechanisms through which it can affect entire food webs.

Model projections suggest a future reduction in the Arctic ice cap and sea ice thickness, along with earlier ice breakup^11–13^. This will certainly affect the timing of key processes such as primary productivity pulses, with potential deleterious consequences for secondary producers^8–10^ that could spread up to higher trophic levels and impact predators such as seabirds (Fig. 3). However, all of these environmental stressors interact in a number of ways, and little is known about the potential for synergetic or antagonistic interactions that may exacerbate or counteract deleterious effects on marine communities^36^. For instance, the greater light availability caused by a reduction in sea ice may increase open-water phytoplankton primary production^37, 38^, thus enhancing food availability. Nonetheless, nutrient limitation driven by the earlier, environmentally-driven pulse of ice algae could ultimately constrain the magnitude of this increase^19, 39^. Changes in community composition may also complicate this picture, as do potential changes in species distribution^6, 7, 17^. Indeed, with a reduction in zooplankton grazers, the high primary production sinks and is exported to the shallow sediments, supporting a large and diverse benthic community that is critical for benthic-feeding marine mammals and seabirds^21, 40^. Top-predators’ breeding parameters, which apparently respond rapidly, sensitively and integratively to environmentally-driven changes in food availability, might therefore become suitable biomonitoring tools for tracing changes in Arctic marine ecosystem functioning^24^.

Due to the high uncertainty inherent to the dynamic and complex marine environments, any precise prediction of the future impacts of climate-driven changes on Arctic food webs in general, and seabirds in particular, would be very speculative. This uncertainty also applies to the spatiotemporal variation in the processes governing sea ice melting. Overall, the timing of ice breakup has slightly advanced during the last few decades^11–13^. However, these trends spatially differ from local to regional scales^7, 20^. Our fine-scale analysis reveals that these trends may be influencing Arctic seabird species differentially according to their foraging range. In particular, the inshore Brünnich’s guillemots, which mainly forage in fjords in Svalbard, are apparently facing a slower advancement in the timing of ice melt compared to the offshore-foraging little auks, whose foraging ranges on the western coast of Spitsbergen are increasingly influenced by the relatively warm and salty Atlantic water^41, 42^. Spatial differences in the magnitude of environmental trends should therefore be considered an additional source of uncertainty in assessments of climate impacts on Arctic marine communities^10^.

The advancement in ice breakup may result in an earlier onset of the pelagic phytoplankton bloom^10^. In addition to its potential impacts on food abundance and nutrient transfer^8, 9^, this advancement may also result in a temporal mismatch between seasonal patterns in food availability and the reproductive requirements of seabirds. Indeed, observed trends in the advancement of melting processes contrasted with seabird phenological responses, as mean hatching dates hold relatively constant throughout the study period (Table 1), like for other Arctic seabirds^14^. The fitness consequences of mismatch processes have been previously reported for a number of northern seabirds. For instance, the timing of reproduction of Atlantic puffins (*Fratercula artica*) is adjusted to food abundance and climate variability^43^, and this timing to some extent affects their reproductive success^28^. Similarly, common murres (*Uria aalge*), Cassin’s auklet (*Ptychoramphus aleuticus*) and rhinoceros auklet (*Cerorhinca monocerata*) tend to match their breeding phenology to the annual peak of abundance in some of their prey, again with temporal mismatch influencing their reproductive performance^44–46^. As expected for species that operate at the upper limit of their energetic capabilities^46–48^, Arctic seabirds should be particularly sensitive to environmental changes affecting the seasonal patterns in resource availability and food abundance^28^.

The warming of the Arctic is leading to changes in sea ice melting phenology, a key driver of primary production and potentially of the reproductive performance of Arctic predators. This is especially concerning considering that warming may accelerate^49^ and that some Arctic seabirds like the Brünnich’s guillemot, are already declining in a large part of their range, including Svalbard^50^. Changes thus far may seem relatively limited but seabirds may be unable to adjust their life cycle to these changes, which may have detrimental consequences on their reproduction and thus population dynamics.

## Methods

### Data collection

Between 2011 and 2016, an average of 103 (range 70–151) and 51 (range 35–66) nests of Brünnich’s guillemots were monitored during the breeding season at Diabasodden (Isfjorden) and Ossian sarsfjellet (Kongsfjorden), respectively (Fig. 1). Data from black-legged kittiwake came from the Grumantbyen colony (Isfjorden), which comprises ca. 40 nests (range 17–59 depending on the year) that were monitored during the 2008–2016 breeding periods. An average of 34 (range 25–45) nests of little auks were monitored during the 2007–2016 breeding periods at the Bjørndalen colony (Isfjorden). Nests were checked 2 to 4 times per week to estimate their phenology schedule and breeding success (hatching success and percentage of chick survival at 15 days after hatching, Table 1). Some Brünnich’s guillemots (n = 103) and black-legged kittiwake (n = 26) were also captured during the chick-rearing period and fitted with a GPS (Global Positioning System) logger (CatTrack 1, Catnip Technologies Ltd., USA; see Supporting Information) in 2012–2015. For little auks, 103 individuals were captured during this period, and ca. 0.3 ml of blood was collected for measurements of carbon isotopic ratios (*δ* ^13^C). All captures, handling, tissue sampling and logger deployments were approved and conducted according to the permits provided by the Governor of Svalbard (project RIS 361) and by the Norwegian Animal Research Authority (NARA/FDU permits # 6293, 6439 and 4154). Birds were handled about 10 m away from their nest, and released after handling, typically after 5 to 15 minutes. The attachment methods for the GPS devices were developed to minimize the impact of the logger on the birds^51^ and have been successfully applied to seabirds previously^52^.

Based on tracking data from Brünnich’s guillemots (43 GPS-retrievals) and black-legged kittiwakes (13 GPS-retrievals), and on the comparison of sea-surface temperature (SST) during the breeding period and blood isotopic ratios (*δ* ^13^C) for little auks, we identified the main foraging areas for each species and colony. The isotopic approach consists of evaluating the temporal covariance between seabird *δ* ^13^C values and SST values on a pixel basis^53^ (see Supporting Information).

The diffuse attenuation coefficient for downwelling irradiance at 490 nm (hereafter KD490 in m^−1^, years 2007 to 2016) was used as an index of light availability within the water column, the ultimate physical factor driving marine productivity at these high latitudes. We used remote sensing data on sea ice extent within defined seabird foraging grounds (km^2^, 2007–2016) to unravel intra-annual trends in sea ice phenology, a proxy for seasonal patterns in sea-ice algae productivity. Finally, CHL-*a* (mg·m^−3^, 2007–2016) was used to examine the seasonal patterns in phytoplankton production after sea ice break-up (see details in Supporting Information).

To frame our results within the current context of climate-driven environmental changes at these latitudes, we used long-term (1979–2012), satellite data on the onset of snowmelt (National Snow and Ice Data Center, Boulder, Colorado USA) to evaluate spatiotemporal changes in the melting phenology. This dataset includes yearly snowmelt onset dates (in Julian days from January, 1^st^) at a 25-km spatial resolution and has been previously considered as a suitable climate proxy in the Arctic sea ice zone^54^.

### Statistical analysis

Whereas information on ice extent is provided on a daily basis, we used weekly composites of KD490 and CHL-*a* to avoid the large spatial gaps of daily images. Data on ice extent, KD490 and CHL-*a* were averaged within the seabirds’ foraging grounds. The smaller spatial coverage for KD490 data prevented us from describing seasonal changes in this parameter within fjords. Non-parametric locally smoothing functions (loess function) were used to remove excessive noise in the original signal.

The annual peaks of sea ice extent and CHL-*a* (in weeks from January 1^st^) were respectively considered as suitable surrogates of the dual peak of primary productivity driving the Arctic bloom: the annual peak of ice algae growing within and beneath the sea ice, and the annual peak of phytoplankton growing in open waters after ice break-up^10, 20^. The timing of these blooms is crucial for the quantity and quality of primary and secondary production^8, 9, 20^. The temporal delay -in weeks- between the annual peaks of sea ice extent and CHL-*a* was therefore considered as an index of the efficiency with which biomass and energy are transferred from primary producers to higher trophic levels. Seabird breeding performance (hatching success and percentage of chick survival at 15 days after hatching) was therefore modeled (linear models) by using estimated temporal lags as the main explanatory variable. Additionally, our models included the “year” as a factor to account for other, unknown drivers of seabirds breeding performance that might vary over time. A three level factor combining the ‘species’ and ‘colony (i.e. Brünnich’s guillemots from Diabasodden – Isfjorden – and Ossian sarsfjellet – Kongsfjorden – and little auks from Bjørndalen) and its interaction with the estimated temporal lags were also considered to account for potential differences in populations’ responses (see details in Supporting Information).

Pixel-based, least squares linear regressions of snow melt onset dates were used for deriving the magnitudes of temporal trends in melting phenology over the past three decades within our study area, thereby providing information about spatial heterogeneity in current climate impacts. Further, the long-term records of average melt onsets within seabirds’ foraging grounds were used to depict the ongoing changes in melting processes (and, potentially, in the productivity patterns) that these seabirds have faced in recent decades.

## Electronic supplementary material

Supporting Information
