# Author Correction: Sea ice phenology and primary productivity pulses shape breeding success in Arctic seabirds

**DOI:** 10.1038/s41598-020-75964-z

**Published:** 2020-11-09

**Authors:** Francisco Ramírez, Arnaud Tarroux, Johanna Hovinen, Joan Navarro, Isabel Afán, Manuela G. Forero, Sébastien Descamps

**Affiliations:** 1 Estación Biológica de Doñana (EBD-CSIC), Sevilla, Spain; 2 Norwegian Polar Institute, Fram Centre, 9296 Tromsø, Norway

Correction to: *Scientific Reports* 10.1038/s41598-017-04775-6, published online 03 July 2017

This Article contains errors in Table 1, where the mean hatching dates and corresponding standard deviations (SD) have been incorrectly listed.

The ‘Mean (SD)’ reported for Species ‘Little auk’, Colony ‘Bjørndalen (Isfjorden)’ change for the date ‘2007, jul-10’,

“(4.3)”

should read:

“(5.6)”

The ‘Mean (SD)’ for Species ‘Little auk’, Colony ‘Bjørndalen (Isfjorden)’ change for the date ‘2008, jul-09’,

“(3.2)”

should read:

“(4.5)”

The ‘Mean (SD)’ for Species ‘Little auk’, Colony ‘Bjørndalen (Isfjorden)’ change for the date ‘2009, jul-12’,

“(2)”

should read:

“(3.5)”

In the same Table 1, the ‘Hatching Dates’ and their associated ‘Mean (SD)’ Species ‘Brünnich’s guillemot’, Colony ‘Diabasodden (Isfjorden)’ change for the year ‘2014′,

“jul-01 (7.9)”

should read:

“jul-07 (3.7)”

The ‘Hatching Dates’ and their associated ‘Mean (SD)’ Species ‘Brünnich’s guillemot’, Colony ‘Diabasodden (Isfjorden)’ change for the year ‘2015′,

“jun-22 (10.7)”

should read:

“jul-03 (5.9)”

Lastly, in the same Table 1, the ‘Hatching Dates’ and their associated ‘Mean (SD)’ Species ‘Brünnich’s guillemot’, Colony ‘Ossian sarsfjellet (Kongsfjorden)’ change for the year 2014.

“jun-29 (6.3)”

should read:

“jul-06 (5.4)”

The ‘Hatching Dates’ and their associated ‘Mean (SD)’ Species ‘Brünnich’s guillemot’, Colony ‘Ossian sarsfjellet (Kongsfjorden)’ change for the year 2015,

“jun-23 (9.2)”

should read:

“jul-02 (7.1)”

The correct Table 1 appears below.

**Table 1:** Summary of the main breeding parameters. Mean hatching date and standard deviation (SD), hatching success and chick survival at 15 days after hatching for the little auk and the Brünnich’s guillemot in the Svalbard archipelago.

Species	Colony	Year	Hatching date		Breeding performance (%)	
			Mean	(SD)	Hatching success	Chick survival
Little auk	Bjørndalen (Isfjorden)	2007	jul-10	(5.6)	88.1	81.0
		2008	jul-09	(4.5)	89.3	78.6
		2009	jul-12	(3.5)	79.5	56.4
		2010	jul-08	(4.4)	100	92.5
		2011	jul-10	(5)	77.8	81.5
		2012	jul-10	(3.5)	91.1	100
		2013	jul-13	(2.7)	87.5	92.9
		2014	jul-10	(2.2)	100	95.2
		2015	jul-08	(2.7)	80	89.5
		2016	jul-06	(1.9)	90	70
Brünnich’s guillemots	Diabasodden (Isfjorden)	2011	jul-03	(3.2)	87.9	75.9
		2012	jul-07	(4.3)	67.4	70.7
		2013	jul-05	(5.5)	69.9	71.8
		2014	jul-07	(3.7)	51.9	76.5
		2015	jul-03	(5.9)	83.1	80.8
		2016	jun-26	3.3	90	77.8
	Ossian sarsfjellet (Kongsfjorden)	2011	jul-04	(5.3)	89.1	54.3
		2012	jul-05	(6.4)	94.1	79.3
		2013	jul-04	(6.5)	85.5	77.1
		2014	jul-06	(5.4)	80.6	87
		2015	jul-02	(7.1)	89.9	91.8
		2016	jul-02	(9.2)	89.4	73.7

