# Supplementary material for: Sea ice phenology and primary productivity pulses shape breeding success in Arctic seabirds
Source: Sci Rep. 2017 Jul 3;7:4500. doi: 10.1038/s41598-017-04775-6 (PMC5495753; doi:10.1038/s41598-017-04775-6)
Supplement: Supplementary file 1 — Supporting Information [file 41598_2017_4775_MOESM1_ESM.pdf]

Supporting Information for:

Sea ice phenology and primary productivity pulses shape breeding success in Arctic seabirds

Francisco Ramírez[1*], Arnaud Tarroux[2], Johanna Hovinen[2], Joan Navarro[1], Isabel Afán[1], Manuela G. Forero[1] and Sébastien Descamps[2]

1. Estación Biológica de Doñana (EBD-CSIC), Sevilla, Spain.

2. Norwegian Polar Institute, Fram Centre, 9296 **Tromsø**, Norway

---

Study area and model species 2

Identification of summer foraging areas for Svalbard Brünnich's guillemots, black-legged kittiwakes and little auks 2

Physical and biological processes driving the productivity cascade 7

Trophic disruptions as a driver of seabird breeding performance: model outputs 9

LITERATURE CITED 11

Study area and model species

The Svalbard archipelago is located within the Barents Sea region. This area is particularly impacted by ocean warming, partly due to the increasing influence of warm Atlantic waters flowing northward in recent years [1,2]. Several seabird species coexist during the breeding season in Svalbard, and the Brünnich's guillemot, black-legged kittiwake and little auk represent some of the most important seabird species in number and in biomass of prey consumed. The inshore-foraging Brünnich's guillemots mainly feed on fish (such as polar cod *Boreogadus saida*) and pelagic crustaceans [3]. They lay a single egg in late May/early June, incubate for about a month and then feed their chick at the nest for approximately three weeks until the end of July/early August. The generalist black-legged kittiwake's diet consists predominantly of fish (e.g. polar cod, capelin *Mallotus villosus*) and marine invertebrates [3,4]. Kittiwake reproduction usually starts in early June and birds typically incubate two eggs for about four weeks. Fledging then occurs in early/mid-August. The offshore-foraging little auk, the smallest species studied, is an exclusively Arctic species that lays a single-egg clutch in early/mid-June. Incubation lasts about a month and fledging generally occurs early August. During summer, little auks feed extensively on *Calanoid* copepods, preferably those found in cold, Arctic water masses near the ice edge such as *Calanus glacialis* [5,6].

Identification of summer foraging areas for Svalbard Brünnich's guillemots, black-legged kittiwakes and little auks

We equipped a total of 129 individuals (n=103 Brünnich's guillemots and 26 black-legged kittiwakes) with GPS-units (CatTrack 1, Catnip Technologies Ltd., Anderson, USA) during the chick-rearing periods of 2012 to 2015 at three different

colonies: Diabasodden (Isfjorden, 2012-2015) and Ossian Sarsfjellet (Kongsfjorden, 2015) in the case of Brünnich's guillemot, and Grumantbyen (Isfjorden, 2013-2014) for black-legged kittiwake. The original plastic packaging was replaced by waterproof heat-shrink tube, for a final mass of ca. 20g, representing 2% and 5% of Brünnich's guillemot and black-legged kittiwake average body mass, respectively (i.e., within the 3-5% limit recommended by most authors [7,8]). We used Tesa tape to attach the GPS units to body feathers on the back of the birds. GPSs were programmed to record locations every 30 seconds when the bird moved and 1 location every 5 or 10 minutes otherwise. Retrievals accounted for 56 individuals, 43 Brünnich's guillemots and 13 black-legged kittiwakes. All locations were projected into Universal Transverse Mercator coordinates (UTM zone 33N) and speed ($km \cdot h^{-1}$) between successive raw positions were computed in R version 3.0.2 [9]. Bird behavior (active foraging vs. others) at each location was categorized on the basis of estimated speeds. Threshold limits of active foraging positions (speed > 3.7 $km \cdot h^{-1}$ and 3.4 $km \cdot h^{-1}$ for Brünnich's guillemot and black-legged kittiwakes, respectively) were estimated through a visual inspection of speed histograms (Figure S1). For black-legged kittiwakes, the main foraging areas were defined as the area covered by the 75% isopleths of bivariate normal kernel utilization distributions computed in ArcGIS10 (ESRI, Redland, USA) using exclusively the active foraging locations. Defined foraging area for black-legged kittiwakes accounted for ca. 14,000 km^2 including most of the fjord and a vast area at the permanently ice-free, western side of Spitsbergen. In the case of Brünnich's guillemot, the main foraging areas were restricted to small areas within the fjords and near the colonies. Due to the lack of remote-sensing products with a sufficient spatial coverage to provide environmental information for these small areas, we considered the

entire fjords for extracting information on oceanographic features relevant for the Brünnich's guillemot.

__Figure S1.__ GPS recorded locations for the Brünnich's guillemots (n=43) and black-legged kittiwakes (n=13). Upper figures show the histograms for estimated speeds between successive locations, along with the speed threshold used for categorizing each location as active foraging vs. others. Lower figures show the active foraging locations upon which the main foraging areas for black-legged kittiwakes are based (see above for details). In the case of Brünnich's guillemot, main foraging areas encompassed small marine areas within fjords and we decided to consider the entire fjords as suitable and meaningful areas for extracting oceanographic information. Map generated with ArcGIS 10.2.1 (ESRI, Redland), www.arcgis.com. Organisational License provided by CSIC.

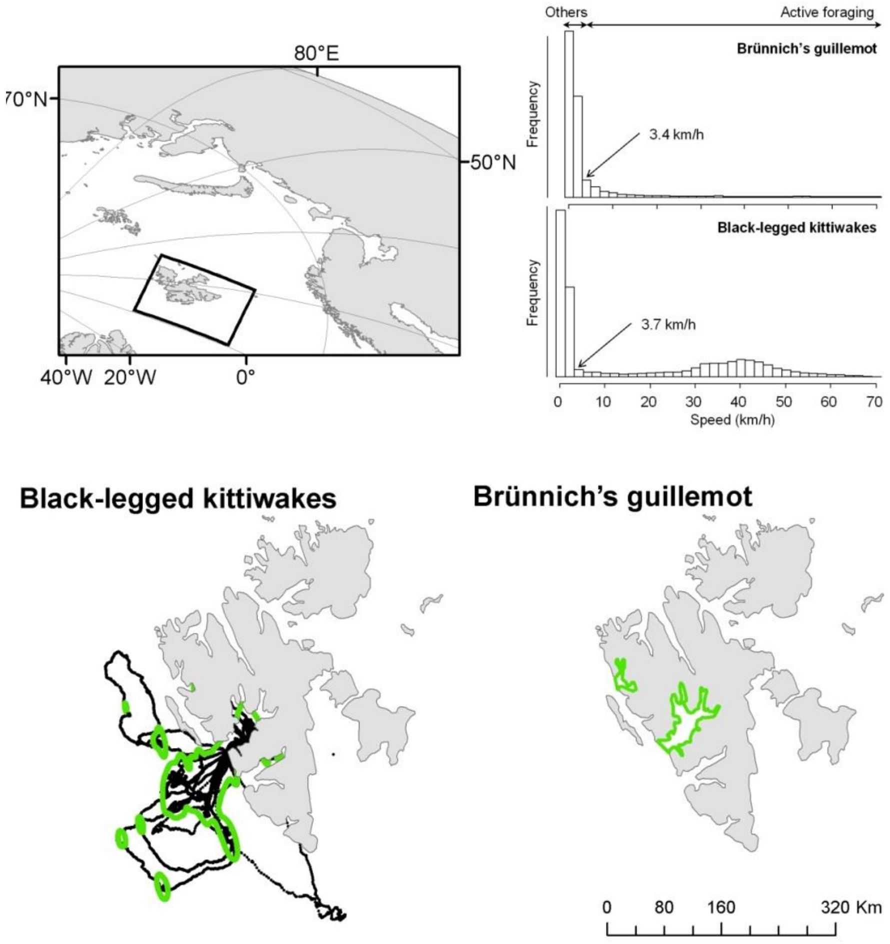

Tracking little auks is logistically complicated due to the small size of the birds. Consequently, little information on foraging tactics for this species is available (but see Welcker et al. [10], Brown et al. [11], Jakubas et al. [12], Hovinen et al. [13]). Little auks tend to

alternate long and short trips for self-feeding and chick provisioning [10,11,14], with longer, self-feeding trips reaching up to 200 km from the colony [11,13]. Following MacKenzie *et al.*[15], we used whole blood δ^{13}C values of adults to estimate the most likely foraging areas for the little auks during long trips. The isotopic composition of carbon in marine organisms is strongly dependent on the fractionation of C during photosynthesis [16]. This fractionation varies inversely with the ratio of cell growth rate to dissolved carbonate concentration [17–19], which, in turn, is intrinsically related to temperature, and varies spatially and temporally across ocean basins [20], leading to spatio-temporal variations in the δ^{13}C values [21]. To determine the likely location of foraging areas for our little auks, we assessed the temporal covariance between blood δ^{13}C values and sea surface temperature (SST) in July (the time-window in which all blood was collected and for which adult blood is likely integrating dietary information), in each one-degree grid square and within a 200 km buffer from their colony at Bjørndalen for the period of 2009-2015 (Figure S2). Areas with the highest correspondence (highest values of Pearson correlation coefficient r) between inter-annual variations in SST and measured δ^{13}C values are suggested as the most likely marine foraging areas during the deposition of blood tissue [15]. This approach considers no interannual changes in the main foraging areas (but see Hovinen *et al.* [13]) and little spatial drift of biomass while energy flows up through the little auks' food web. Considered foraging areas for the little auk encompass one-degree pixels with Pearson correlation coefficients (r) > 0.35. With a total surface of ~19,000 km^2, we identified two potential areas located at the North and South of Spitsbergen, respectively. These areas fit with the idea that little auks preferentially target cold and highly-productive waters around the south of Spitsbergen [22,23] and/or areas close to the limits of their foraging range and the ice edge where their staple prey, the copepods of the genus *Calanus* spp., tend to aggregate [24].

Monthly SST was sourced online as a netCDF product (NOAA_OI_SST_V2), provided by the NOAA/OAR/ESRL PSD, Boulder, Colorado, USA (http://www.esrl.noaa.gov/psd/; accessed on November 2015).

Figure S2. *Estimated foraging area for little auks breeding in Bjørndalen (red dot). Main foraging areas were estimated by assessing the covariance between inter-annual variations in seabird' $\delta^{13}C$ values and that for SST on a pixel basis within a 200 km buffer from the colony. Considered foraging area encompasses one-degree pixels with Pearson correlation coefficients (r) > 0.35. The upper plot shows the temporal covariance between blood $\delta^{13}C$ values and SST within defined foraging grounds. Maps include information on ice extent (National Snow and Ice Data Center, Boulder, Colorado USA). Background colors pinpoint the seasonal ice zone, based on daily images of ice extent for the 2006-2016 period (National Snow and Ice Data Center, Boulder, Colorado USA). Map generated with ArcGIS 10.2.1 (ESRI, Redland), www.arcgis.com. Organisational License provided by CSIC.*

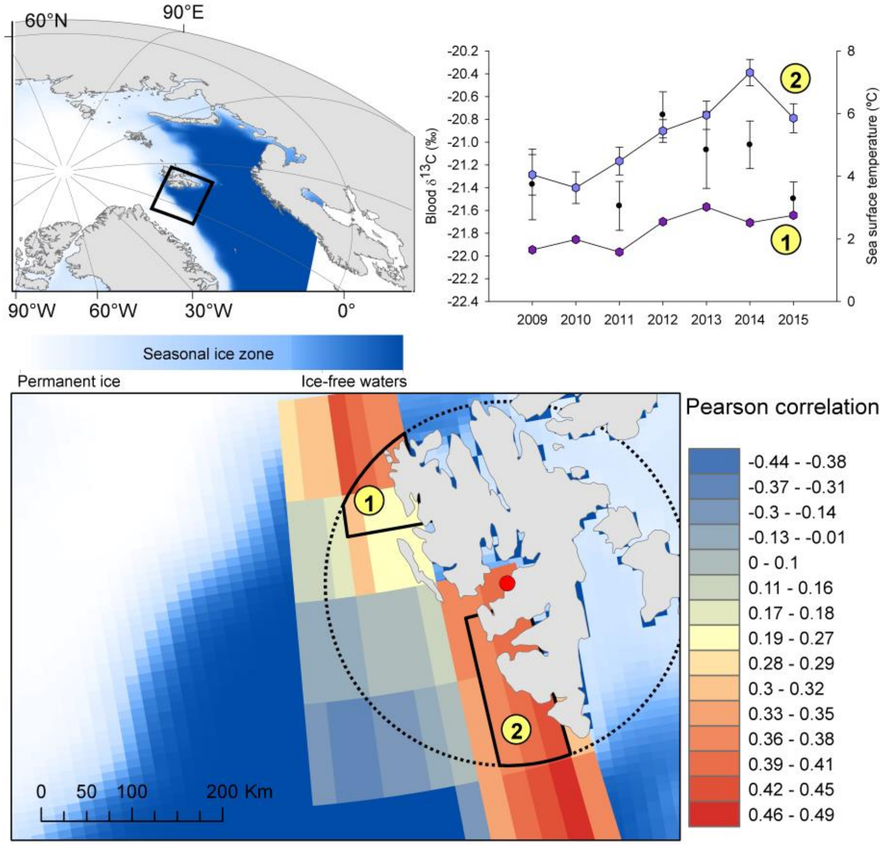

Physical and biological drivers of marine productivity

Marine productivity at the seasonal ice zones of Svalbard is largely driven by the timing of the two consecutive pulses of marine autotrophs that form the base of the Arctic food webs [24,25]. In turn, these two productive periods are constrained by the darkness that persists for four months during the winter season and the sea ice cover. Sea-ice algae begin to grow within and underneath the sea ice in early spring, concurrently with increasing day lengths and with the start of the snow and ice melting processes. Pelagic algal bloom, in contrast, normally occurs in open waters after ice breakup (but see Ji *et al.* [26]), and constitutes the only peak in productivity that is reliably measureable through satellite remote-sensing derived sea surface chlorophyll-*a* concentration records (CHL-*a*, in mg m^{-3}).

Here we obtained satellite remote sensing records on ice extent, light availability within the water column and CHL-*a*, the main physical and biological drivers of the two main productivity pulses. Daily information on global ice extent (2007 to 2016) was sourced online as an ESRI-supported shapefile product (Multisensor Analyzed Sea Ice Extent – Northern Hemisphere; MASIE-NH) from the National Snow and Ice Data Center, National Oceanic and Atmospheric Administration, Boulder, Colorado, USA (https://nsidc.org/data/masie/; accessed on December 2016). The diffuse attenuation coefficient for downwelling irradiance at 490nm (hereafter KD490 in m^{-1}, years 2007 to 2016) was used as an index of light penetration into the water column. KD490 is an indicator of the turbidity of the water column; that is, how visible light in the blue-green part of the spectrum penetrates the water column. It is directly related to the presence of scattering particles in the water column and equivalent to more traditional direct measurements such as the 'Secchi depth' [27]. KD490 was sourced online (http://oceancolor.gsfc.nasa.gov/cms/, accessed in December 2016) as eight-day

composite, level 3 Hierarchical Data Format (HDF) from Aqua MODIS sensors at a 0.041667º horizontal resolution. We finally used two different variables informing about CHL-*a*. For the little auk, we used weekly information on CHL-*a* (2007-2016, accessed in December 2016) from a L4 reanalysis by the European Space Agency (ESA). This product uses regional OC5ci chlorophyll algorithms to compute surface CHL-a ($mg \cdot m^{-3}$) at 1 km resolution using data on Ocean Colour CCI Remote Sensing Reflectance (merged, bias –corrected Rrs) [28,29]. This information was sourced online from MyOcean – Copernicus website: http://marine.copernicus.eu/ (accessed on October 2016). Due to its insufficient spatial coverage for unravelling intra-annual patterns in CHL-*a* within Svalbard fjords, we additionally used a modelled product providing weekly mean global information on CHL-*a* at a 0.5°·spatial resolution for the 2011-2016 period (sourced online from MyOcean – Copernicus website: http://marine.copernicus.eu/; accessed on December 2016). This product was produced by Mercator Ocean (Tolouse, France) and uses an offline coupling between a global biogeochemical model PISCES and the Mercator Ocean global 0.25º systems (see details in MyOcean – Copernicus).

High-temporal resolution satellite records were used to evaluate seasonal patterns in these main physical and biological drivers. The annual peak of sea ice was used as a surrogate for the onset of sea-ice melting and, consequently, the timing of the annual bloom of sea ice algae [30]. Seasonal patterns in CHL-*a* were used to derive open-water, pelagic algal blooms. The temporal lag between these two main pulses of primary productivity was considered as the main environmental factor potentially driving biomass and energy transfer from primary producers up to top predators [24–26]. Estimated temporal lags were used, therefore, to evaluate the role of these environmentally-driven, bottom-up processes affecting food abundance in explaining

interannual differences in the breeding performance of high Arctic top-predators such as seabirds.

Trophic disruptions driving seabird breeding performance: model outputs

Linear models were used to investigate the role of environmentally-driven, interannual changes in the timing of the two marine productivity pulses as a potential driver of seabird breeding performance, in terms of hatching success and chick survival. In addition to our estimates of temporal lags between the two productivity pulses, our models included the "year" (to account for other, unknown drivers of seabirds breeding performance that might vary over time), a three-level factor that combined species and colony (i.e. Brünnich's guillemots from Diabasodden – Isfjorden – and Ossian sarsfjellet – Kongsfjorden – and little auks from Bjørndalen – Isfjorden –) and its interaction with the temporal lags (to account for differences among species and localities). Although hatching success and chick survival for different species and colonies were recorded during the same time-period, Pearson's r were relatively low for all combinations of time series (Table S1), thus pointing to their independence and suggesting they could be all included in a single model without incurring in pseudoreplication. For hatching success, only the three-level, Species-colony factor showed a significant effect (Table S2 and Fig. 3). Contrastingly, the model for chick survival showed significant effects of the Temporal lag and the Species-colony factor, thus suggesting similar responses (slopes) for all species and localities (Table S3 and Fig. 3). Overall, the observed trend suggested that chick survival decreased as the temporal lag between the two consecutive pulses of marine productivity increased. When fitting the linear regressions independently for species and colonies, we found

that the effect of the Temporal lag remained negative for all species and colonies, albeit not being statistically significant for Brünnich's guillemots from Diabasodden (Isfjorden) and only nearly statistically significant for this latter species in Ossian Sarsfjellet (Kongsfjorden) (Table S4).

While uncertainty is inherent in marine ecological research the challenge is to implement scientifically sound approaches that will help identifying key issues for marine conservation and that are based on available data. Our study provides such an example by combining remote sensing data with in situ breeding success observations. Our results suggest that, despite some variation among species and even colonies, seabirds' breeding performance is partially driven by the efficiency with which biomass and energy are transferred from primary producers to higher trophic levels. This hypothesis is supported by the well-documented role of sea ice cover as a driver of secondary production [24,25].

Table S1. Pearson's r for all combinations of hatching success and chick survival time series. Correlations were statistically non-significant for combinations ($p>0.05$). A nearly statistically significant correlation was observed for the little auk and Brünnich's guillemots (Isfjorden) hatching success time series ($t = -2.5426$, $df = 4$, p-value = 0.0638)

	Little auk	Brünnich's guillemots (Isfjorden)	Brünnich's guillemots (Kongsfjorden)
**Hatching success**			
Little auk	-	-0.7859807	-0.4967034
Brünnich's guillemots (Isfjorden)		-	0.5594697
Brünnich's guillemots (Kongsfjorden)			-
**Chick survival**			
Little auk	-	-0.5152137	0.4655076
Brünnich's guillemots (Isfjorden)		-	0.2370399
Brünnich's guillemots (Kongsfjorden)			-

Table S2. Output of the model fitted for hatching success.

Main effects:	F	df	p-value
Species-colony	6.02	2	0.037
Temporal lag	2.23	1	0.186
Year (factor)	1.46	9	0.331
Temporal lag:Species-colony	3.32	2	0.107

Estimates:			95% Conf. Interval	
	Estimate	Std. Error	2.5 %	97.5 %
Intercept (ref. level: little auks -Bjørndalen)	101.98	9.33	79.16	124.79
Temporal lag	-2.78	1.01	-5.24	-0.31
Species-colony				
Brünnich's guillemots (Isfjorden)	-7.36	19.25	-54.47	39.74
Brünnich's guillemots (Kongsfjorden)	-13.63	12.29	-43.71	16.44
Year				
2008	-1.59	11.14	-28.84	25.67
2009	-0.28	11.50	-28.42	27.85
2010	11.9	11.09	-15.24	39.04
2011	9.29	10.09	-15.41	33.98
2012	3.28	9.52	-20.02	26.59
2013	0.29	9.61	-23.23	23.82
2014	-2.55	9.63	-26.11	21.00
2015	3.00	10.84	-23.52	29.53
2016	26.1	11.68	-2.47	54.67
Temporal lag:Species-colony				
lag:Brünnich's guillemots (Isfjorden)	-0.24	2.24	-5.73	5.25
lag:Brünnich's guillemots (Kongsfjorden)	2.50	1.29	-0.67	5.67

Table S3. Output of the model fitted for chick survival.

Main effects:	F	df	p-value	
Species-colony	8.31	2	0.019	
Temporal lag	27.78	1	0.002	
Year (factor)	0.66	6	0.686	
Temporal lag:Species-colony	0.52	2	0.620	
**Estimates:**			**95% Conf. Interval**	
	**Estimate**	**Std. Error**	**2.5 %**	**97.5 %**
Intercept (ref. level: little auks -Bjørndalen)	102.57	7.42	84.41	120.73
Temporal lag	-2.01	0.80	-3.98	-0.05
Species-colony				
Brünnich's guillemots (Isfjorden)	-25.27	15.32	-62.76	12.22
Brünnich's guillemots (Kongsfjorden)	-6.90	9.78	-30.84	17.04
Year				
2011	-2.99	8.03	-22.65	16.67
2012	2.62	7.58	-15.93	21.16
2013	-3.28	7.65	-22.01	15.45
2014	2.82	7.66	-15.93	21.56
2015	4.76	8.63	-16.35	25.87
2016	0.33	9.29	-22.41	23.07
Temporal lag:Species-colony				
lag:Brünnich's guillemots (Isfjorden)	1.73	1.79	-2.64	6.10
lag:Brünnich's guillemots (Kongsfjorden)	0.39	1.03	-2.14	2.91

Table S4. Output of the separate regressions fitted for chick survival.

					95% Conf. Interval	
	**t-value**	**p-value**	**Estimate**	**Std. Error**	**2.5%**	**97.5%**
**Little auk**						
Intercept	30.25	0.000	103.48	3.42	94.68	112.27
Temporal lag	-4.96	0.004	-2.06	0.41	-3.12	-0.99
**Brünnich's guillemot**						
Intercept	18.10	0.000	91.12	5.04	79.73	102.51
Temporal lag	-3.17	0.011	-1.46	0.46	-2.51	-0.42
**Isfjorden**						
Intercept	14.15	0.000	81.63	5.77	65.61	97.65
Temporal lag	-1.09	0.338	-0.70	0.64	-2.48	1.08
**Kongsfjorden**						
Intercept	11.84	0.001	99.34	8.39	72.63	126.04
Temporal lag	-2.88	0.064	-1.88	0.65	-3.96	0.20

References

1. Moline, M. A. *et al.* High latitude changes in ice dynamics and their impact on polar marine ecosystems. *Ann. N. Y. Acad. Sci.* **134,** 267–319 (2008).
2. Pavlov, A. K. *et al.* Warming of Atlantic Water in two west Spitsbergen fjords over the last century (1912–2009). *Polar Res.* **32,** (2013).
3. Anker-Nilssen, T. *et al.* The status of marine birds breeding in the Barents Sea region. (2000).
4. Coulson, J. *The Kittiwake*. (T & AD Poyser, 2011).
5. Karnovsky, N. J., Kwaniewski, S., Wsawski, J. M., Walkusz, W. & BeszczyskaMller, A. Foraging behavior of little auks in a heterogeneous environment. *Mar. Ecol. Prog. Ser.* **253,** 289–303 (2003).
6. Kwasniewski, S. *et al.* The impact of different hydrographic conditions and zooplankton communities on provisioning Little Auks along the West coast of Spitsbergen. *Prog. Oceanogr.* **87,** 72–82 (2010).
7. Phillips, R. A., Xavier, J. C. & Croxall, J. P. Effects of Satellite Transmitters on Albatrosses and Petrels. *The Auk* **120,** 1082–1090 (2003).
8. Passos, C., Navarro, J., Giudici, A. & González-Solís, J. Effects of an extra mass on the pelagic behaviour of a seabird. *The Auk* **127,** 100–107 (2010).
9. R Core Team. *R: A language and environment for statistical computing*. (R Foundation for Statistical Computing, 2013).
10. Welcker, J. *et al.* Flexibility in the bimodal foraging strategy of a high Arctic alcid, the little auk *Alle alle*. *J. Avian Biol.* **40,** 388–399 (2009).
11. Brown, Z. W., Welcker, J., Harding, A. M. A., Walkusz, W. & Karnovsky, N. J. Divergent diving behavior during short and long trips of a bimodal forager, the little auk Alle alle. *J. Avian Biol.* **43,** 215–226 (2012).

12. Jakubas, D. *et al.* Foraging closer to the colony leads to faster growth in little auks. *Mar. Ecol. Prog. Ser.* **489,** 263–278 (2013).
13. Hovinen, J. *et al.* At-sea distribution of foraging little auks relative to physical factors and food supply. *Mar. Ecol. Prog. Ser.* **503,** 263–277 (2014).
14. Steen, H., Vogedes, D., Broms, F., Falk-Petersen, S. & Berge, J. Little auks (Alle alle) breeding in a High Arctic fjord system: bimodal foraging strategies as a response to poor food quality? *Polar Res.* **26,** 118–125 (2007).
15. MacKenzie, K. M. *et al.* Locations of marine animals revealed by carbon isotopes. *Sci. Rep.* **1,** (2011).
16. Goericke, R. & Fry, B. Variations of marine plankton δ13C with latitude, temperature, and dissolved CO2 in the world ocean. *Glob. Biogeochem. Cycles* **8,** 85–90 (1994).
17. Laws, E. A., Popp, B. N., Bidigare, R. R., Kennicutt, M. C. & Macko, S. A. Dependence of phytoplankton carbon isotopic composition on growth rate and [CO2)aq: Theoretical considerations and experimental results. *Geochim. Cosmochim. Acta* **59,** 1131–1138 (1995).
18. Laws, E. A., Bidigare, R. R. & Popp, B. N. Effect of growth rate and CO2 concentration on carbon isotopic fractionation by the marine diatom Phaeodactylum tricornutum. *Limnol. Oceanogr.* **42,** 1552–1560 (1997).
19. Tagliabue, A. & Bopp, L. Towards understanding global variability in ocean carbon-13. *Glob. Biogeochem. Cycles* **22,** GB1025 (2008).
20. Richardson, A. J. & Schoeman, D. S. Climate Impact on Plankton Ecosystems in the Northeast Atlantic. *Science* **305,** 1609–1612 (2004).

21. Barnes, C. & Jennings, J. T. B., Simon. Environmental correlates of large-scale spatial variation in the δ13C of marine animals. *Estuar. Coast. Shelf Sci.* **81,** 368–374 (2009).

22. Svendsen, H. *et al.* The physical environment of Kongsfjorden–Krossfjorden, an Arctic fjord system in Svalbard. *Polar Res.* **21,** 133–166 (2002).

23. Blanchet, M.-A., Lydersen, C., Ims, R. A. & Kovacs, K. M. Seasonal, Oceanographic and Atmospheric Drivers of Diving Behaviour in a Temperate Seal Species Living in the High Arctic. *PLOS ONE* **10,** e0132686 (2015).

24. Søreide, J. E., Leu, E., Berge, J., Graeve, M. & Falk-Petersen, S. Timing of blooms, algal food quality and Calanus glacialis reproduction and growth in a changing Arctic. *Glob. Change Biol.* **16,** 3154–3163 (2010).

25. Leu, E., Søreide, J. E., Hessen, D. O., Falk-Petersen, S. & Berge, J. Consequences of changing sea-ice cover for primary and secondary producers in the European Arctic shelf seas: Timing, quantity, and quality. *Prog. Oceanogr.* **90,** 18–32 (2011).

26. Ji, R., Jin, M. & Varpe, Ø. Sea ice phenology and timing of primary production pulses in the Arctic Ocean. *Glob. Change Biol.* **19,** 734–741 (2013).

27. Austin, R. W. & Petzold, T. J. in *Oceanography from Space* (ed. Gower, J. F. R.) 239–256 (Springer US, 1981).

28. Gohin, F. *et al.* Towards a better assessment of the ecological status of coastal waters using satellite-derived chlorophyll-a concentrations. *Remote Sens. Environ.* **112,** 3329–3340 (2008).

29. Hu, C., Lee, Z. & Franz, B. Chlorophyll aalgorithms for oligotrophic oceans: A novel approach based on three-band reflectance difference. *J. Geophys. Res. Oceans* **117,** (2012).

30. Wassmann, P. Arctic marine ecosystems in an era of rapid climate change. *Prog. Oceanogr.* **90,** 1–17 (2011).
